# Organic–inorganic hybrid hexa­chlorido­stannate(IV) with 2-methyl­imidazo[1,5-*a*]pyridin-2-ium cation

**DOI:** 10.1107/S2056989023000324

**Published:** 2023-01-19

**Authors:** Olga Yu. Vassilyeva, Elena A. Buvaylo, Vladimir N. Kokozay, Alexandre N. Sobolev

**Affiliations:** a Department of Chemistry, Taras Shevchenko National University of Kyiv, 64/13 Volodymyrska Street, Kyiv 01601, Ukraine; b School of Molecular Sciences, M310, the University of Western Australia, 35 Stirling Highway, Perth, 6009, W.A., Australia; University of Kentucky, USA

**Keywords:** crystal structure, hybrid salt, tin(IV), 2-pyridine­carbaldehyde

## Abstract

The hybrid salt bis­(2-methyl­imidazo[1,5-*a*]pyridin-2-ium) hexa­chlorido­stannate(IV), (C_8_H_9_N_2_)_2_[SnCl_6_], crystallizes in the monoclinic space group *P*2_1_/*n* with the asymmetric unit containing an Sn_0.5_Cl_3_ fragment (Sn site symmetry  ) and one organic cation. The five- and six-membered rings in the cation are nearly coplanar; bond lengths in the pyridinium ring of the fused core are as expected; the C—N/C bond distances in the imidazolium entity fall in the range 1.337 (5)–1.401 (5) Å. The octa­hedral SnCl_6_^2–^ dianion is almost undistorted with the Sn–Cl distances varying from 2.4255 (9) to 2.4881 (8) Å and the *cis* Cl—Sn—Cl angles approaching 90°. In the crystal, π-stacked chains of cations and loosely packed SnCl_6_^2–^ dianions form separate sheets alternating parallel to (101). Most of the numerous C—H⋯Cl—Sn contacts between the organic and inorganic counterparts with the H⋯Cl distances above the van der Waals contact limit of 2.85 Å are considered a result of crystal packing.

## Abstract 2

The asymmetric unit of the title compound, (C_8_H_9_N_2_)_2_[SnCl_6_], contains one cation in a general position and one-half of the dianion situated on an inversion centre. The octa­hedral SnCl_6_^2–^ dianion is almost undistorted. The crystal structure can be seen as an arrangement of alternating organic and inorganic layers with little support from C—H⋯Cl—Sn contacts.

## 1. Chemical context

Organic–inorganic hybrid perovskites that combine discrete organic cations and rigid metal halide architectures have been considered promising materials for diverse optoelectronic applications: solar cells, light-emitting diodes, photodetectors, spintronics (Gan *et al.*, 2021 ▸; Li *et al.*, 2021 ▸). Most of the materials reported to date are based on Pb^II^, Sb^III^, Bi^III^ and Cd^II^ halides (Saparov & Mitzi, 2016 ▸), whose widespread application is restrained by the potential toxicity. Being in the same main group of metal atoms that Pb belongs to, Sn forms hybrid halide perovskites with similar electronic properties, which are more friendly to the environment. At the same time, the aforementioned hybrid systems suffer from high water permeability and low thermal stability, the issues being largely related to the volatility of small organic cations (Leijtens *et al.*, 2015 ▸). The stability of hybrid perovskites can be improved by introducing larger organic cations and lowering the dimensionality of the octa­hedral halometallate frameworks (Zhang *et al.*, 2016 ▸; Leblanc *et al.*, 2019 ▸). Moreover, functional organic cations are a valuable tool for introducing useful properties into the hybrid structure. For example, the use of the photoactive zwitterion viologen *N*,*N*′-4,4′-bipyridiniodipropionate (CV) afforded the formation of the covalently bonded pillared layered bromo­plumbate, [Pb_3_Br_6_(CV)]_*n*_, showing high thermal stability in air and a remarkable increase of capacitance after photoinduced electron transfer (Sun *et al.*, 2019 ▸). Mono-periodic hybrid lead halides incorporating optically active protonated 1,3-bis­(4-pyrid­yl)-propane cations exhibit dual-light emissions combined of higher energy blue and lower energy yellow light spectra, which were attributed to the individual contributions of the organic and inorganic components (Sun *et al.*, 2021 ▸). 
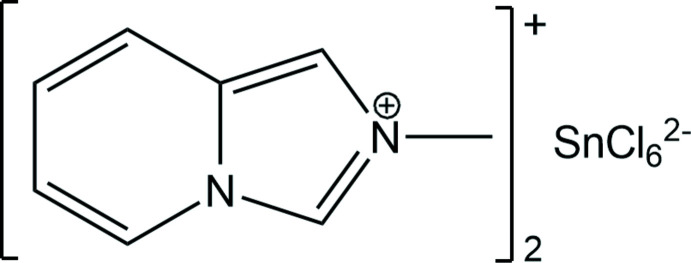


Multiple advantages of the organic–inorganic hybrid materials inspire the huge appeal in exploring other kinds of low-dimensional metal halide compounds templated by functional aromatic cations. Fine-tuning of the electronic structure and optoelectronic properties of the metal halide hybrids, which depend, among other things, on the anionic speciation and halogen ratio, can be achieved by mixing halide ligands in self-assembled organic–inorganic systems (Rogers *et al.*, 2019 ▸; Askar *et al.*, 2018 ▸).

Pursuing our research on hybrid halometalates incorporating substituted imidazo[1,5-*a*]pyridinium cations (Buvaylo *et al.*, 2015 ▸; Vassilyeva *et al.*, 2019 ▸; 2020 ▸; 2021 ▸), we attempted the synthesis of a hybrid tin mixed halide with 2-methyl­imidazo[1,5-*a*]pyridinium, *L* ^+^, a product of the oxidative cyclo­condensation between 2-pyridine­carbaldehyde (2-PCA), formaldehyde and CH_3_NH_2_. One necessary component of the reaction is acid, which is introduced as a hydro­halide adduct of the amine (Vassilyeva *et al.*, 2020 ▸). Following the method of preparation used to obtain mixed-halide Zn^II^ and Cd^II^ tetra­halometalates (Cl/I, Br/Cl) with *L* ^+^ (Vassilyeva *et al.*, 2022 ▸), SnCl_2_·2H_2_O was reacted with the solution of *L* ^+^ formed *in situ* using 2-PCA, formaldehyde and CH_3_NH_2_·HBr. The isolated product was crystallographically characterized as [*L*]_2_[SnCl_6_], (I); the detrimental oxidation of Sn^II^ to Sn^IV^ appeared unavoidable leading to the formation of ubiquitous hexa­chlorido­stannate(IV) dianion. Herein, the synthesis, structural analysis and spectroscopic characterization of (I) are reported.

## 2. Structural commentary

**Figure 1:**
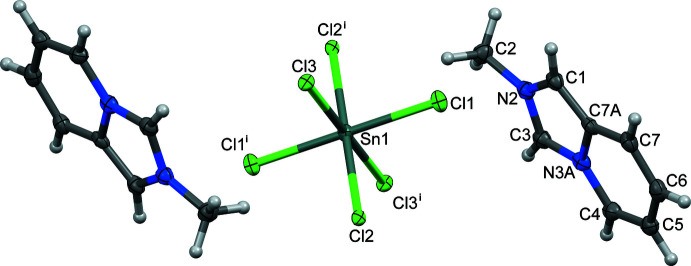
Mol­ecular structure of (I) with atom labelling showing 50% displacement ellipsoids. [Symmetry code: (i) −*x* + 1, −*y* + 1, −*z* + 1.]

The title hybrid salt, with formula (C_8_H_9_N_2_)_2_[SnCl_6_], crystallizes in the monoclinic space group *P*2_1_/*n*. The asymmetric unit consists of an Sn_0.5_Cl_3_ fragment (Sn site symmetry 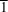 ) and 2-methyl­imidazo[1,5-*a*]pyridinium cation, as shown in Fig. 1 ▸. The structural configuration of the cation is similar to those of other 2-methyl­imidazo[1,5-*a*]pyridinium hybrid salts (C_8_H_9_N_2_)_2_[ZnCl_4_] (GOTHAB; Vassilyeva *et al.*, 2020 ▸) and (C_8_H_9_N_2_)_2_[CdCl_4_] (GOTJAD; Vassilyeva *et al.*, 2021 ▸). Bond lengths in the pyridinium ring of the fused core are as expected; the C—N/C bond distances in the imidazolium entity fall in the range 1.337 (5)–1.401 (5) Å; N2 and N3*A* atoms are planar with the sums of three angles being equal to 360°. The almost coplanar five- and six-membered rings in the cation show the dihedral angle between them of 1.6 (2)°. The octa­hedral SnCl_6_^2–^ dianion in (I) is almost undistorted with the Sn—Cl distances varying from 2.4255 (9) to 2.4881 (8) Å and the *cis* Cl—Sn—Cl angles approaching 90° (Table 1 ▸). The geometric parameters of the dianion are normal and comparable to those of similar structure types.

## 3. Supra­molecular features

**Figure 2:**
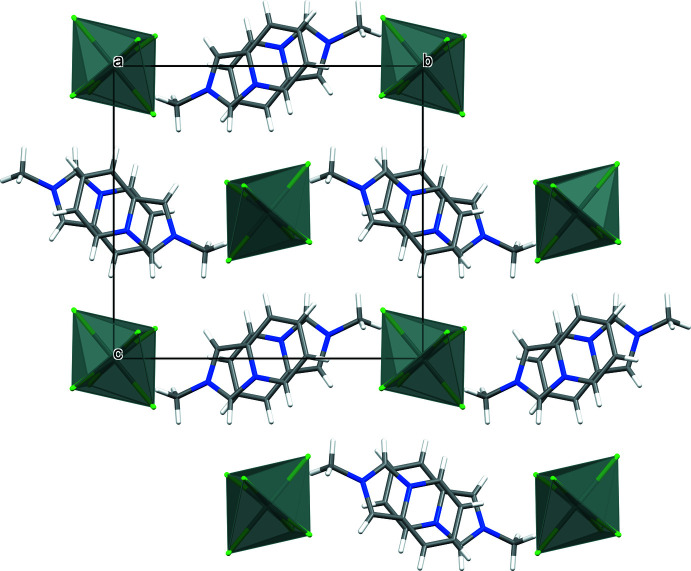
Projection of the crystal packing of (I) on the *bc* plane showing organic and inorganic sheets alternating parallel to the (101) plane.

**Figure 3:**
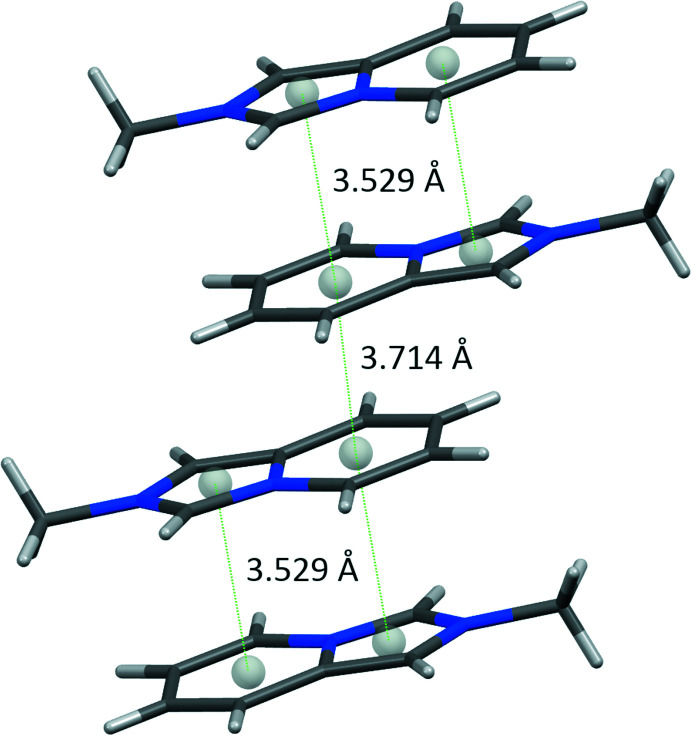
Fragment of the π-stacked chain built of pairs of *L* ^+^ cations of (I).

In the crystal, cationic and anionic sheets alternate lying parallel to (101) (Fig. 2 ▸). In the sheets, pairs of centrosymmetically related *trans*-oriented *L* ^+^ cations demonstrate offset 10πe–10πe stacking with a centroid–centroid distance of 3.530 (2) Å (Fig. 3 ▸). The pairs further form π-bonded chains with a distance of 3.713 (2) Å between neighbouring pyridin­ium ring centroids. In the anion sheet, loose packing of SnCl_6_^2–^ dianions that are identically stacked one above the other with the shortest Sn–Cl⋯Cl–Sn distance being 4.4433 (12) Å, results in a closest separation of 7.7926 (1) Å between the metal atoms. The hybrid salt lacks classical hydrogen-bonding inter­actions but shows a variety of C—H⋯Cl—Sn contacts between the organic and inorganic counterparts (Table 2 ▸), a feature common to hybrid chloro­metalates with nitro­gen-containing aromatic cations (Coleman *et al.*, 2013 ▸). Most of these contacts are longer than the van der Waals contact limit of 2.85 Å (Cl) (Mantina *et al.*, 2009 ▸) and can be considered a result of crystal packing.

## 4. Database survey

A search of the Cambridge Structural Database (CSD, Version 5.42; Groom *et al.*, 2016 ▸) for structures including substituted imidazo[1,5-*a*]pyridinium cations gave 53 salts with about a half (23) reported by our research group. The latter comprise organic–inorganic hybrids with the *L* ^+^ cation or its derivatives [2-methyl-3-(pyridin-2-yl)imidazo[1,5-*a*]pyridin-2-ium and 2,2′-(ethane-1,2-diyl)bis(imidazo[1,5-*a*]pyridin-2-ium)] counterbalanced by transition and main-group (Mn, Cu, Zn, Cd, Pb) halometalates. The other comp­ounds in the CSD with cations similar to *L* ^+^ are mostly organic salts with the imidazo[1,5-*a*]pyridinium core having various substituents in the rings. Perchlorate NAKNET (Mishra, *et al.*, 2005 ▸) and hexa­fluoro­phosphate DIWYEP (Kriechbaum, *et al.*, 2014 ▸), which bear methyl­phenyl and di­methyl­phenyl substituents, respectively, in place of the methyl group in *L* ^+^ are the most closely related. Structures of main group halometalates with substituted imidazo[1,5-*a*]pyridinium cations are limited to a few examples such as bis­[2-(6-methyl­pyridin-2-yl)imidazo[1,5-*a*]pyridin-2-ium] di­chloro­gold tetra­chloro­gold (SUWVIR; Nandy *et al.*, 2016 ▸) and 2-(2-ammonio­cyclo­hex­yl)-3-(pyridin-2-yl)imidazo[1,5-*a*]pyridin-2-ium hexa­bromo­tell­urium aceto­nitrile solvate (TEVVIB; Vasudevan *et al.*, 2012 ▸).

Within the variety of 279 crystal structures in the CSD comprising [SnCl_6_]^2–^ dianions, the latter are mostly highly symmetrical being associated with special positions. The structures including organic counterparts can be seen as an arrangement of alternating organic and inorganic layers supported by hydrogen bonds of the N–H⋯Cl type in the case of protonated N-containing cations. An organic–inorganic hybrid compound with the structure most similar to that of the title compound is, for example, monoclinic bis­[1-(prop-2-en-1-yl)-1*H*-imidazol-3-ium] hexa­chlorido­stannate(IV), in space group *P*2_1_/*n*, with layers formed by isolated [SnCl_6_]^2–^ octa­hedra and (C_6_H_9_N_2_)^+^ organic cations, which propagate along the *a*-axis direction at *y* = 0 and *y* = 1/2 (Ferjani, 2020 ▸).

## 5. Synthesis and crystallization

Synthesis of [*L*]_2_[SnCl_6_] (I). Solid CH_3_NH_2_·HBr (0.45 g, 4 mmol) was added to the warm formaldehyde solution prepared by dissolving paraform (0.13 g, 4.5 mmol) in boiling deionized water (10 ml) in a 50 ml conical flask. The solution was stirred vigorously for 1 h at room temperature and filtered. On the following day, 2-PCA (0.38 ml, 4 mmol) was introduced into the flask under stirring, followed by the addition of SnCl_2_·2H_2_O (0.22 g, 1 mmol) dissolved in ethanol (10 ml) in 30 min. The solution was kept magnetically stirred at room temperature for another hour, then filtered to remove Sn(OH)_2_ and allowed to evaporate. It was diluted with methanol (5 ml) since it was thickening. Pale-yellow needles of (I) suitable for X-ray crystallography formed over three months after successive addition of ^*i*^PrOH (5 ml). The crystals were filtered off, washed with diethyl ether and dried in air. Yield: 12% (based on Sn). FT–IR (ν, cm^−1^): 3422*br*, 3122*vs*, 3092*vs*, 3050*vs*, 3014, 2956, 2914, 1654, 1566, 1544, 1454, 1374, 1352, 1328, 1258, 1222, 1148vs, 1130, 1038, 986, 920, 789*vs*, 764, 742, 666, 624*vs*, 498, 468, 434. ^1^H NMR (400 MHz, DMSO-*d*_6_): δ (ppm) 9.83 (*s*, 1H, H_C3_), 8.69 (*d*, 1H, *J* = 6.8 Hz, H_C4_), 8.24 (*s*, 1H, H_C1_), 7.82 (*d*, 1H, *J* = 9.2 Hz, H_C7_), 7.22 (*t*, 1H, *J* = 8.2 Hz, H_C5_), 7.13 (*t*, 1H, *J* = 6.7 Hz, H_C6_), 4.27 (*s*, 3H, CH_3_). Analysis calculated for C_16_H_18_N_4_SnCl_6_ (597.73): C 32.15; H 3.04; N 9.37%. Found: C 32.40; H 2.88; N 9.19%.

## 6. Refinement

Crystal data, data collection and structure refinement details are summarized in Table 3 ▸. All hydrogen atoms were included in calculated positions and refined using a riding model with isotropic displacement parameters based on those of the parent atom (C—H = 0.95 Å, *U*_iso_(H) = 1.2*U*_eq_C for CH, C—H = 0.98 Å, *U*_iso_(H) = 1.5*U*_eq_C for CH_3_). Anisotropic displacement parameters were employed for the non-hydrogen atoms.

## Supplementary Material

Crystal structure: contains datablock(s) I, global. DOI: 10.1107/S2056989023000324/pk2675sup1.cif

Structure factors: contains datablock(s) I. DOI: 10.1107/S2056989023000324/pk2675Isup2.hkl

CCDC reference: 2235690

Additional supporting information:  crystallographic information; 3D view; checkCIF report

## Figures and Tables

**Table 1:** Selected geometric parameters (Å, °)

Sn1—Cl1	2.4255 (9)	Sn1—Cl2	2.4881 (8)
Sn1—Cl3	2.4777 (8)		
			
Cl1^i^—Sn1—Cl3	89.72 (3)	Cl3—Sn1—Cl2^i^	90.59 (3)
Cl1—Sn1—Cl3	90.28 (3)	Cl1—Sn1—Cl2	89.80 (3)
Cl1—Sn1—Cl2^i^	90.20 (3)	Cl3—Sn1—Cl2	89.41 (3)

Symmetry code: (i) 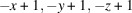 .

**Table 2:** Hydrogen-bond geometry (Å, °)

*D*—H⋯*A*	*D*—H	H⋯*A*	*D*⋯*A*	*D*—H⋯*A*
C5—H5⋯Cl3^ii^	0.95	2.97	3.614 (5)	126
C3—H3⋯Cl2^iii^	0.95	2.65	3.549 (4)	158
C3—H3⋯Cl1^iii^	0.95	2.91	3.483 (4)	120
C4—H4⋯Cl3^iii^	0.95	2.96	3.473 (4)	115
C7—H7⋯Cl3^iv^	0.95	2.96	3.746 (5)	141
C7—H7⋯Cl1^iv^	0.95	2.91	3.605 (4)	131
C2—H2*A*⋯Cl1^iii^	0.98	2.86	3.668 (5)	140
C2—H2*B*⋯Cl2^v^	0.98	2.91	3.658 (5)	134
C2—H2*C*⋯Cl2^i^	0.98	2.87	3.804 (4)	161
C2—H2*C*⋯Cl1	0.98	2.96	3.615 (4)	125

Symmetry codes: (i) 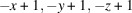 ; (ii) 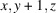 ; (iii) 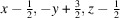 ; (iv) 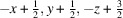 ; (v) 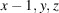 .

**Table 3:** Experimental details

Crystal data	
Chemical formula	(C_8_H_9_N_2_)_2_[SnCl_6_]
*M*_r_	597.73
Crystal system, space group	Monoclinic, *P*2_1_/*n*
Temperature (K)	100
*a*, *b*, *c* (Å)	7.7926 (1), 12.1425 (1), 11.7114 (1)
β (°)	101.082 (1)
*V* (Å^3^)	1087.49 (2)
*Z*	2
Radiation type	Mo *K*α
μ (mm^−1^)	1.92
Crystal size (mm)	0.38 × 0.20 × 0.13

Data collection	
Diffractometer	Oxford Diffraction Gemini-R Ultra
Absorption correction	Analytical [*CrysAlis PRO* (Rigaku OD, 2016 ▸); analytical numeric absorption correction using a multifaceted crystal model based on expressions derived by Clark & Reid (1995 ▸)]
*T*_min_, *T*_max_	0.618, 0.802
No. of measured, independent and observed [*I* > 2σ(*I*)] reflections	18730, 2215, 2108
*R*_int_	0.035
(sin θ/λ)_max_ (Å^−1^)	0.625

Refinement	
*R*[*F* ^2^ > 2σ(*F* ^2^)], *wR*(*F* ^2^), *S*	0.034, 0.098, 1.00
No. of reflections	2215
No. of parameters	125
H-atom treatment	H-atom parameters constrained
Δρ_max_, Δρ_min_ (e Å^−3^)	1.54, −1.15

Computer programs: *CrysAlis PRO* (Rigaku OD, 2016 ▸), *SHELXT2015/1* (Sheldrick, 2015*a* ▸), *SHELXL2019/2* (Sheldrick, 2015*b* ▸), *Mercury* (Macrae *et al.*, 2020 ▸) and *WinGX* (Farrugia, 2012 ▸).

## supplementary crystallographic information

### Crystal data

**Table:**

(C_8_H_9_N_2_)_2_[SnCl_6_]	*F*(000) = 588
*M**_r_* = 597.73	*D*_x_ = 1.825 Mg m^−^^3^
Monoclinic, *P*2_1_/*n*	Mo *K*α radiation, λ = 0.71073 Å
*a* = 7.7926 (1) Å	Cell parameters from 17399 reflections
*b* = 12.1425 (1) Å	θ = 2.4–37.5°
*c* = 11.7114 (1) Å	µ = 1.92 mm^−^^1^
β = 101.082 (1)°	*T* = 100 K
*V* = 1087.49 (2) Å^3^	Needle, pale yellow
*Z* = 2	0.38 × 0.20 × 0.13 mm

### Data collection

**Table:**

Oxford Diffraction Gemini-R Ultra diffractometer	2215 independent reflections
Radiation source: fine-focus sealed X-ray tube, Enhance (Mo) X-ray Source	2108 reflections with *I* > 2σ(*I*)
Graphite monochromator	*R*_int_ = 0.035
ω scans	θ_max_ = 26.4°, θ_min_ = 2.4°
Absorption correction: analytical [CrysAlisPro (Rigaku OD, 2016); analytical numeric absorption correction using a multifaceted crystal model based on expressions derived by Clark & Reid (1995)]	*h* = −9→9
*T*_min_ = 0.618, *T*_max_ = 0.802	*k* = −15→15
18730 measured reflections	*l* = −14→14

### Refinement

**Table:**

Refinement on *F*^2^	0 restraints
Least-squares matrix: full	Hydrogen site location: inferred from neighbouring sites
*R*[*F*^2^ > 2σ(*F*^2^)] = 0.034	H-atom parameters constrained
*wR*(*F*^2^) = 0.098	*w* = 1/[σ^2^(*F*_o_^2^) + (0.050*P*)^2^ + 6.970*P*] where *P* = (*F*_o_^2^ + 2*F*_c_^2^)/3
*S* = 1.00	(Δ/σ)_max_ < 0.001
2215 reflections	Δρ_max_ = 1.54 e Å^−^^3^
125 parameters	Δρ_min_ = −1.15 e Å^−^^3^

### Special details

**Table:**

Geometry. All esds (except the esd in the dihedral angle between two l.s. planes) are estimated using the full covariance matrix. The cell esds are taken into account individually in the estimation of esds in distances, angles and torsion angles; correlations between esds in cell parameters are only used when they are defined by crystal symmetry. An approximate (isotropic) treatment of cell esds is used for estimating esds involving l.s. planes.

### Fractional atomic coordinates and isotropic or equivalent isotropic displacement parameters (Å^2^)

**Table:**

	*x*	*y*	*z*	*U*_iso_*/*U*_eq_	
Sn1	0.500000	0.500000	0.500000	0.01497 (14)	
Cl1	0.35555 (12)	0.63407 (8)	0.60266 (8)	0.0195 (2)	
Cl2	0.78853 (11)	0.57935 (7)	0.59047 (7)	0.01241 (19)	
Cl3	0.52226 (11)	0.37088 (7)	0.66602 (7)	0.0141 (2)	
C1	0.0945 (5)	0.8366 (3)	0.5329 (4)	0.0206 (8)	
H1	0.048748	0.798625	0.591356	0.025*	
N2	0.0816 (4)	0.8046 (3)	0.4195 (3)	0.0199 (7)	
C2	−0.0015 (6)	0.7034 (4)	0.3679 (4)	0.0250 (9)	
H2A	−0.023505	0.709472	0.282891	0.038*	
H2B	−0.112456	0.692324	0.393919	0.038*	
H2C	0.075990	0.640636	0.392353	0.038*	
N3A	0.2249 (4)	0.9583 (3)	0.4373 (3)	0.0190 (7)	
C3	0.1607 (5)	0.8781 (3)	0.3620 (4)	0.0206 (8)	
H3	0.169920	0.874245	0.282421	0.025*	
C4	0.3127 (5)	1.0545 (3)	0.4176 (4)	0.0222 (8)	
H4	0.334519	1.070978	0.342328	0.027*	
C5	0.3661 (5)	1.1237 (4)	0.5076 (4)	0.0232 (9)	
H5	0.425896	1.189613	0.495717	0.028*	
C6	0.3333 (6)	1.0986 (4)	0.6211 (4)	0.0251 (9)	
H6	0.373638	1.147621	0.683721	0.030*	
C7A	0.1860 (5)	0.9340 (3)	0.5465 (3)	0.0185 (8)	
C7	0.2459 (6)	1.0064 (3)	0.6405 (4)	0.0214 (9)	
H7	0.225017	0.990431	0.716016	0.026*	

### Atomic displacement parameters (Å^2^)

**Table:**

	*U*^11^	*U*^22^	*U*^33^	*U*^12^	*U*^13^	*U*^23^
Sn1	0.0167 (2)	0.0148 (2)	0.0133 (2)	0.00138 (12)	0.00244 (14)	0.00014 (12)
Cl1	0.0212 (5)	0.0206 (5)	0.0163 (4)	0.0061 (3)	0.0027 (3)	−0.0021 (3)
Cl2	0.0146 (4)	0.0125 (4)	0.0100 (4)	−0.0003 (3)	0.0023 (3)	−0.0006 (3)
Cl3	0.0182 (4)	0.0135 (4)	0.0104 (4)	−0.0010 (3)	0.0023 (3)	0.0003 (3)
C1	0.0206 (19)	0.021 (2)	0.0205 (19)	0.0058 (15)	0.0052 (15)	0.0039 (15)
N2	0.0186 (16)	0.0202 (17)	0.0210 (17)	0.0036 (13)	0.0041 (13)	0.0026 (13)
C2	0.024 (2)	0.023 (2)	0.027 (2)	−0.0007 (16)	0.0010 (17)	−0.0001 (17)
N3A	0.0171 (16)	0.0225 (18)	0.0173 (16)	0.0037 (13)	0.0027 (13)	0.0041 (13)
C3	0.0182 (18)	0.023 (2)	0.0201 (19)	0.0039 (15)	0.0032 (15)	0.0035 (16)
C4	0.0195 (19)	0.022 (2)	0.026 (2)	0.0030 (16)	0.0054 (16)	0.0068 (16)
C5	0.0193 (19)	0.021 (2)	0.029 (2)	0.0039 (15)	0.0043 (16)	0.0036 (17)
C6	0.023 (2)	0.025 (2)	0.027 (2)	0.0074 (17)	0.0017 (17)	−0.0025 (17)
C7A	0.0157 (18)	0.0229 (19)	0.0178 (19)	0.0077 (15)	0.0057 (14)	0.0051 (15)
C7	0.022 (2)	0.023 (2)	0.020 (2)	0.0074 (15)	0.0052 (16)	0.0010 (15)

### Geometric parameters (Å, º)

**Table:**

Sn1—Cl1^i^	2.4255 (9)	C2—H2C	0.9800
Sn1—Cl1	2.4255 (9)	N3A—C3	1.345 (6)
Sn1—Cl3	2.4777 (8)	N3A—C4	1.395 (5)
Sn1—Cl3^i^	2.4778 (8)	N3A—C7A	1.401 (5)
Sn1—Cl2^i^	2.4881 (8)	C3—H3	0.9500
Sn1—Cl2	2.4881 (8)	C4—C5	1.350 (6)
C1—N2	1.369 (5)	C4—H4	0.9500
C1—C7A	1.373 (6)	C5—C6	1.433 (6)
C1—H1	0.9500	C5—H5	0.9500
N2—C3	1.337 (5)	C6—C7	1.352 (6)
N2—C2	1.465 (5)	C6—H6	0.9500
C2—H2A	0.9800	C7A—C7	1.416 (6)
C2—H2B	0.9800	C7—H7	0.9500
			
Cl1^i^—Sn1—Cl1	180.0	N2—C2—H2C	109.5
Cl1^i^—Sn1—Cl3	89.72 (3)	H2A—C2—H2C	109.5
Cl1—Sn1—Cl3	90.28 (3)	H2B—C2—H2C	109.5
Cl1^i^—Sn1—Cl3^i^	90.28 (3)	C3—N3A—C4	129.1 (4)
Cl1—Sn1—Cl3^i^	89.72 (3)	C3—N3A—C7A	109.1 (3)
Cl3—Sn1—Cl3^i^	180.00 (2)	C4—N3A—C7A	121.8 (4)
Cl1^i^—Sn1—Cl2^i^	89.80 (3)	N2—C3—N3A	107.6 (4)
Cl1—Sn1—Cl2^i^	90.20 (3)	N2—C3—H3	126.2
Cl3—Sn1—Cl2^i^	90.59 (3)	N3A—C3—H3	126.2
Cl3^i^—Sn1—Cl2^i^	89.41 (3)	C5—C4—N3A	118.6 (4)
Cl1^i^—Sn1—Cl2	90.20 (3)	C5—C4—H4	120.7
Cl1—Sn1—Cl2	89.80 (3)	N3A—C4—H4	120.7
Cl3—Sn1—Cl2	89.41 (3)	C4—C5—C6	120.6 (4)
Cl3^i^—Sn1—Cl2	90.59 (3)	C4—C5—H5	119.7
Cl2^i^—Sn1—Cl2	180.0	C6—C5—H5	119.7
N2—C1—C7A	107.2 (4)	C7—C6—C5	121.1 (4)
N2—C1—H1	126.4	C7—C6—H6	119.4
C7A—C1—H1	126.4	C5—C6—H6	119.4
C3—N2—C1	110.1 (4)	C1—C7A—N3A	105.9 (4)
C3—N2—C2	124.2 (4)	C1—C7A—C7	135.3 (4)
C1—N2—C2	125.7 (4)	N3A—C7A—C7	118.8 (4)
N2—C2—H2A	109.5	C6—C7—C7A	119.0 (4)
N2—C2—H2B	109.5	C6—C7—H7	120.5
H2A—C2—H2B	109.5	C7A—C7—H7	120.5
			
C7A—C1—N2—C3	−0.2 (4)	N2—C1—C7A—N3A	0.5 (4)
C7A—C1—N2—C2	178.1 (4)	N2—C1—C7A—C7	−178.1 (4)
C1—N2—C3—N3A	−0.2 (4)	C3—N3A—C7A—C1	−0.6 (4)
C2—N2—C3—N3A	−178.5 (3)	C4—N3A—C7A—C1	177.7 (3)
C4—N3A—C3—N2	−177.7 (4)	C3—N3A—C7A—C7	178.2 (3)
C7A—N3A—C3—N2	0.5 (4)	C4—N3A—C7A—C7	−3.4 (6)
C3—N3A—C4—C5	−179.8 (4)	C5—C6—C7—C7A	−0.1 (6)
C7A—N3A—C4—C5	2.2 (6)	C1—C7A—C7—C6	−179.2 (4)
N3A—C4—C5—C6	0.1 (6)	N3A—C7A—C7—C6	2.3 (6)
C4—C5—C6—C7	−1.1 (6)		

Symmetry code: (i) −*x*+1, −*y*+1, −*z*+1.

### Hydrogen-bond geometry (Å, º)

**Table:**

*D*—H···*A*	*D*—H	H···*A*	*D*···*A*	*D*—H···*A*
C5—H5···Cl3^ii^	0.95	2.97	3.614 (5)	126
C3—H3···Cl2^iii^	0.95	2.65	3.549 (4)	158
C3—H3···Cl1^iii^	0.95	2.91	3.483 (4)	120
C4—H4···Cl3^iii^	0.95	2.96	3.473 (4)	115
C7—H7···Cl3^iv^	0.95	2.96	3.746 (5)	141
C7—H7···Cl1^iv^	0.95	2.91	3.605 (4)	131
C2—H2*A*···Cl1^iii^	0.98	2.86	3.668 (5)	140
C2—H2*B*···Cl2^v^	0.98	2.91	3.658 (5)	134
C2—H2*C*···Cl2^i^	0.98	2.87	3.804 (4)	161
C2—H2*C*···Cl1	0.98	2.96	3.615 (4)	125

Symmetry codes: (i) −*x*+1, −*y*+1, −*z*+1; (ii) *x*, *y*+1, *z*; (iii) *x*−1/2, −*y*+3/2, *z*−1/2; (iv) −*x*+1/2, *y*+1/2, −*z*+3/2; (v) *x*−1, *y*, *z*.
